# Poison Apple-like Fruit Ingestion: A Case Report and Review of Manchineel Fruit Toxicity

**DOI:** 10.3390/ijerph23081062

**Published:** 2026-08-16

**Authors:** Amanda K. Hempel, Rahel Zewude, Michael Klowak, Tahyreem Shahid, Judith Joshi, Zhongtian Shao, Andrea K. Boggild

**Affiliations:** 1 Temerty Faculty of Medicine, University of Toronto, Toronto, ON M5S 1A8, Canada; 2 Institute of Medical Science, University of Toronto, Toronto, ON M5S 3H2, Canada; 3 School of Medicine, Trinity College Dublin, D02 PN40 Dublin, Ireland; 4 Tropical Disease Unit, Toronto General Hospital, Toronto, ON M5G 2C4, Canada

**Keywords:** environmental health, food safety, intoxication, plant toxin, toxicity, toxidrome

## Abstract

**Highlights:**

**Public health relevance—How does this work relate to a public health issue?**

- Exposure to the sap and fruit of the manchineel tree may cause a toxidrome of potentially severe dermatologic, gastrointestinal, and hemodynamic morbidity.
- Manchineel trees and their fruits are not inherently recognizable as toxic by those visiting endemic areas and those unfamiliar with the clinical consequences of exposure.

**Public health significance—Why is this work of significance to public health?**

- Manchineel toxicity is a morbid and potentially fatal condition that is entirely preventable through appropriate public health messaging and education.
- Manchineel toxicity is costly from an individual patient perspective and also from a health systems perspective, particularly given the distribution of manchineel trees in low- and middle-income countries of the Caribbean, Central America, and West Africa.

**Public health implications—What are the key implications or messages for practitioners, policy makers, and/or researchers in public health?**

- Beaches lined by manchineel trees should be clearly marked as such with unequivocal signage regarding the toxicity of manchineel sap, fruit, and bark. In addition to clear signage at sites of manchineel growth, awareness regarding the risk of manchineel toxicity should be raised through other public health messaging and risk communication platforms.
- Clinicians should become familiar with the symptoms and signs of manchineel toxicity including blistering dermatitis, gastroenteritis, and hemodynamic effects.

**Abstract:**

Manchineel dermatitis, caused by the sap of the manchineel tree native to the coastlands of the Caribbean, West Africa, and Central America, has received limited attention in the literature despite significant morbidity. The toxic sap causes a burning, blistering rash and conjunctivitis, while ingestion of the fruit may lead to oropharyngeal pain, hemodynamic instability, and gastrointestinal distress. Tourists unfamiliar with the manchineel tree may be exposed through exposure to the tree’s sap, contaminated rainwater, or ingestion of the apple-like fruit. We herein present a case of a patient who experienced prolonged symptoms of manchineel toxicity after ingestion of the manchineel fruit for several weeks. Antihistamines, corticosteroids, and a PPI were ineffective. The symptoms eventually resolved after discarding clothing and towels that had come in contact with the tree, raising the possibility that toxins may linger on fabrics. Travellers to regions in the Caribbean or Central America are at particular risk for toxicity from the Manchineel tree if unfamiliar with the associated dangers and should be counselled to avoid contact with the tree or ingesting any unfamiliar fruit on the ground. When exposures do occur, it may be reasonable to dispose of potentially contaminated fabrics to avoid recurrent exposures, particularly if recurrent symptoms are observed, as in our patient. We further contextualize manchineel toxicity within a comparative review of select tropical foodborne intoxications of plant origin (ackee, lychee, cassava, papain) and marine origin (ciguatera, scombroid, neurotoxic and diarrhetic shellfish poisonings, tetrodotoxin), distinguishing manchineel’s local irritant mechanism from the systemic toxidromes of these comparators and underscoring the absence of a specific antidote.

## 1. Introduction

The manchineel tree, found throughout the coastlands of the Caribbean and Central America in particular, is considered one of the most dangerous trees in the world. The sap contains toxins that cause severe dermatitis and conjunctivitis, while ingestion of the fruit can lead to oropharyngeal injuries and hemodynamic disturbances [1,2,3,4]. Although this severe toxicity has been known for hundreds of years, there is a paucity of literature describing the pathophysiology, clinical presentations, or treatment for this intoxication syndrome. Tourists unfamiliar with the tree may be exposed, as they do not recognize the risk both the tree and its fruit pose. In accordance with CARE guidance, we herein describe a case of a Canadian traveller who ingested the manchineel fruit and experienced prolonged and treatment-refractory symptoms of intoxication. In this review, we further contextualize manchineel toxicity within a comparative analysis of select tropical foodborne intoxications of plant origin (Table 1; ackee, lychee, cassava, papain) and marine origin (Table 2; ciguatera, scombroid, neurotoxic and diarrhetic shellfish poisonings, tetrodotoxin), distinguishing manchineel’s local irritant mechanism from the systemic toxidromes of these comparators and underscoring the absence of a specific antidote.

## 2. Case Description

A woman in her 70s from Canada presented to our unit with illness following travel to Costa Rica for the purpose of tourism. On day 0 of her illness, while at the beach, she was sitting under a tree when she picked up two ‘apples’ from the sand and ate them. Within thirty minutes, she experienced a bitter taste, a whitened colour, and a burning sensation of her tongue, along with swelling of her lips and oropharynx. She had diaphoresis, palpitations, and a single episode of diarrhea. She presented later that same day to a local clinic, where she was given antihistamines and intravenous fluids with some improvement in her symptoms. She then learned the ‘apples’ were fruit from the toxic manchineel tree.

On day 1, she was seen locally by a gastroenterologist for ongoing burning and bitter taste, and she received further antihistamines, prednisone 5 mg daily, and a proton pump inhibitor (PPI) for 5 days. A gastroscopy was not performed. Her symptoms improved gradually over days 1 to 3. However, from day 3 through day 14, comprising the remainder of her two-week travel, she experienced intermittent episodes of bitter taste, oral paresthesias, lip swelling, palpitations, and diarrhea despite a second five-day course of antihistamines, prednisone 5 mg daily, and a PPI.

On day 14, she returned to Canada but continued to have intermittent mild oral swelling, paresthesias, and bitter taste with associated palpitations. Her symptoms improved with oral cetirizine. Approximately one month following return (approximate day 45 post-exposure), she was referred to our tropical medicine unit, where a physical exam identified no perioral swelling or lesions, and investigations found a normal complete blood count (CBC), electrolyte profile, creatinine, liver enzymes, TSH, and serum vitamin B12 level. Blood cultures that were drawn at consultation due to reported diaphoresis were negative, as were serologies for Hepatitis A, B and C, *Strongyloides*, and syphilis. A transthoracic echocardiogram and 48 h Holter monitor were also unremarkable. She was counselled to use cetirizine as needed. It was recommended that she discard all clothing and towels that had been in contact with the tree as a precautionary measure. At follow-up one month later (approximate day 75), her symptoms had reportedly resolved after these steps were taken, and she was able to stop cetirizine. Subsequent follow-up at 3 months (approximate day 135) revealed no further recurrence of the intoxication symptoms.

## 3. Discussion and Review

The manchineel tree, *Hippomane mancinella*, belongs to the Euphorbiaceae family and produces one of the most potent tree toxins known [2]. Damage to any part of the tree can result in the release of white, milky sap (latex), which is rich in irritants. Skin contact with this latex causes painful, blistering dermatitis. Eye contact with the sap or smoke from tree burning can cause keratoconjunctivitis and temporary blindness, and ingestion of fruits from the tree can cause immediate oropharyngeal inflammation followed by gastrointestinal distress [5]. Reports of toxic ingestions and dermatitis exist as far back as the 16th century [6]. The spherical green fruit, sometimes called a “beach apple,” was referred to as the “manzanilla de la Muerte” or “little apple of death” by the Spanish, while the Kalinago Indigenous people from the lesser Antilles (formerly known as Island Caribs) used the milky caustic sap to poison arrow tips [1,2,7]. Even rainwater dropping from the tree is known to cause caustic burns of the skin and mucous membranes [3,4].

Despite this well-described toxicity, there is a paucity of literature on the etiology of the toxic effects of the manchineel tree, and a dearth of information forewarning travelers of the perils of ingestion or contact with the tree and its fruit. Given the manifold and systemic effects on the human body, it is likely that there is more than one toxin in the sap and fruit. Hippomanin A and diterpene esters have been isolated from manchineel and provoke a profound irritant dermatitis [3,7,8,9,10]. Hippomanin A is a hydrolysable tannin (ellagintannin) isolated from the ethanolic extract of *Hippomane mancinella* [9]. Hydrolysable tannins are known for their ability to produce astringent/burning sensations in the oropharynx. Interaction of tannins with mucosal protein can result in aggregation and precipitation of proteins, which may lead to immediate oral irritation [11]. Diterpene esters are potent irritants found across the Euphorbiaceae family. Specifically, diterpenes esters such as tiglianes and ingenanes are structurally related to phorbol esters, which drive inflammation by activating protein kinase C (PKC). Activation of PKC promotes downstream cytokine release, immune cell recruitment, vasodilation, and vascular injury. These effects may correspond to the pain, erythema, blistering dermatitis, and intense mucosal inflammation seen after *Hippomane mancinella* exposure. The esterification and solubility of diterpene esters modulate the tissue penetration and toxic potential of these compounds. This may contribute to the ability of these compounds to result in a range of symptoms after exposure [12]. One remote study in 1955 also isolated physostigmine from the fruit; however, a later investigation failed to corroborate this finding. Screening of harvested fruit by thin layer chromatography and by liquid chromatography with high-resolution mass spectrometry detected none, and the original finding is attributed to the low specificity of the colour reaction used, with ellagic tannins proposed as a plausible source of the false positive [2,13].

The manchineel tree itself grows above the high-water mark on narrow coastal belts of the Caribbean, the island of Tobago, Central America, Florida, the US Virgin Islands, and the West African coast [2,7,14]. It does not tolerate frost, which confines it to the tropics and, at the northern limit of its range, to the southern tip of Florida, where it is protected under state law. The species is not globally threatened, but it has been subject to extensive felling in parts of the Caribbean due to toxicity. The incidents of manchineel tree toxicity center around tourists and migrants who do not recognize the tree and its toxic properties. The removal of manchineel trees carries an ecological cost as the trees stabilize coastal soils to limit beach erosion. Clear signage combined with public education is the more appropriate response since there is no specific prophylaxis/antidote available for manchineel poisoning and public health control must coexist with conservation measures [2,3]. The trees grow to under 25 m and produce a small green fruit resembling a crab apple [2,3,6]. Exposures come in a number of forms, such as inadvertent skin or mucosal contact or from cutting down trees [3]. Although often marked with an ‘X’ in South Florida, Caribbean islands, and some coastal regions in Central and South America to warn of danger, tourists unfamiliar with the tree may use it for shelter, and the fruit’s appearance and appetizing smell occasionally lead to ingestion. Markings tend to be concentrated in tourist areas such as resorts, beaches, and parks. These trees are often unmarked in more undeveloped areas. Since there is no single standardized system for marking these trees, tree marking is often dependent on local authorities and managers [2,3,4,7]. Several cases have been reported of persons sheltering under the tree during rain experiencing severe symptoms from extensive exposure to rainwater dripping off the tree containing dissolved toxins [2,3,4].

The symptoms have primarily been described in case reports and series. The most commonly described symptom is a severe irritant contact dermatitis or chemical burn from contact with the tree sap or associated rainwater [1,3,4,15]. The rash is accompanied by burning and pruritus that may progress to swaths of erythema and blisters, known as manchineel dermatitis [1]. Ocular injury with severe conjunctivitis has also been described [3,15,16]. Depending on the extent of exposure, these symptoms may start within hours and last hours to days [3]. No antidote or antitoxin exists for manchineel fruit poisoning. Treatment primarily involves rigorous cleansing with clean water to remove the toxin and supportive management for symptomatic relief, but corticosteroids occasionally have been used, as we saw with our patient [3]. Ocular exposure to manchineel tree sap or fruits requires irrigation with copious amounts of clean water followed by a prompt ophthalmology evaluation. While no definitive guideline exists for the treatment of manchineel ocular injuries, in the largest published case series of manchineel keratoconjunctivitis, all patients were treated with lavage, cycloplegics and topical antibiotics to prevent secondary bacterial infections [3]. The skin and ocular manifestations of manchineel toxicity are similar to the toxic effects caused by contact with the sap from other Euphorbia plants. In general, the clinical presentation of dermatitis caused by Euphorbiaceae is self-resolving and depends on the amount and duration of sap exposure. A range of effects including pain, itching, erythema, edema, vesicles, bullae, ulcers, and necrosis have been previously reported [17,18,19,20,21]. A review of 7 cases found that ocular exposure to Euphorbia plant sap resulted in pain, loss of visual acuity, keratoconjunctivitis and uveitis [22].

Consumption of the fruit has led to a spectrum of clinical manifestations ranging from asymptomatic exposure to death. The largest published experience is a retrospective series of 97 ingestions reported to French Poison Control Centres, in which 97% of patients were symptomatic, with a mean of 2.3 symptoms each. The most frequent were oropharyngeal pain (68%), abdominal pain (42%), diarrhea (37%), oropharyngeal irritation (32%) and vomiting (20%), and severity was graded as absent in 5%, mild in 84% and moderate in 10% [2]. Treatment in these cases was purely supportive. A few cases have reported gastritis and esophagitis with ulcerative lesions and bleeding that can be fatal [2,7]. Twenty-nine of the 97 patients were tourists, and case numbers peaked in January and July, corresponding to the main tourist seasons in the French West Indies [2]. The quantity of manchineel fruit ingested appears to influence severity. Eleven patients in that series ate more than one fruit, and this group was significantly older than those eating one or fewer (mean 55.6 versus 33.9 years) and reported significantly more symptoms (3.4 versus 2.2), with a non-significant trend toward greater severity [2]. Cardiovascular effects have been described in the same context, with sinus bradycardia reported in two older adults who had each eaten two or more fruits, persisting for ten days in one case and requiring pacemaker insertion [2,23]. On this basis, the authors of the series recommend cardiac monitoring for several hours after ingestion of multiple fruits, and reserve upper gastrointestinal endoscopy for ingestion of several fruits, oropharyngeal edema or ulceration, hematemesis, or persistent retrosternal or epigastric pain [2].

The mechanism behind the bradyarrhythmia effects triggered by manchineel fruit poisoning is still speculative. It is still unclear which compounds are responsible for these effects. Older studies have proposed that manchineel fruits may contain physostigmine (anticholinesterase alkaloid), which could increase the availability of acetylcholine at the sinoatrial (SA) node and trigger parasympathomimetic bradycardia [2,13]. However, more recent evidence contradicts this theory. For instance, one case with bradycardia in the French Poison Control Centres retrospective series completed a chemical analysis of harvested manchineel fruits and did not detect the presence of physostigmine [2]. Phorbol esters and diterpene compounds, which are thought to be responsible for the mucosal effects of manchineel fruit poisoning, are potent activators of protein kinase C and inflammatory pathways. It is possible that these compounds may cause cardiovascular effects through direct cardiotoxic and other indirect mechanisms. For instance, it is possible that these compounds could cause severe mucosal irritation, which could trigger a vagal reflex leading to bradyarrhythmia and hypotension. However, the association between phorbol esters and diterpene compounds with the cardiovascular manifestations of manchineel fruit poisoning has not been tested experimentally [12].

Similar to many of the cases reported in the literature, our patient was a tourist who did not recognize the dangers of the manchineel tree and ingested what she thought were apples on the beach. Her acute symptoms of oropharyngeal burning, mucosal swelling and diarrhea were among those most frequently reported in other series and case reports. She also falls within the higher-risk group defined by that series, being in her 70s and having eaten two fruits, and both published patients with documented bradycardia were likewise older adults who had eaten two or more fruits [2,23]. She was managed supportively, as were all patients in that series [2]. Upper gastrointestinal endoscopy was not performed, but applying the criteria proposed by the series, her ingestion of two fruits together with oropharyngeal swelling might have warranted it, although she was assessed abroad and her symptoms were improving at the time. No electrocardiogram was obtained during the acute episode despite her reported palpitations, and cardiac monitoring after ingestion of more than one fruit is now recommended [2].

Our patient’s course differed from these reports in two respects. No cardiac abnormality was demonstrated, since her palpitations were subjective and both the transthoracic echocardiogram and 48 h Holter monitor, performed several weeks after ingestion, were unremarkable, in contrast to the documented and sustained bradycardia in the two published cases. Her symptoms also relapsed over several weeks. Protracted effects are not themselves unprecedented, as bradycardia persisted for ten days in one report and for at least two weeks in another, but a relapsing oral and mucosal course of this kind has not been previously described [2,23]. These recurrences resolved only after she discarded clothing and a towel that had been in contact with the tree. Although it is possible she had protracted symptoms from the original exposure, this temporality also raises the possibility that toxins from the tree remained on the fabric and led to repeated lower-dose mucosal exposures through hand-to-mouth transfer. Several features of these manchineel fruit compounds make persistence on fabric plausible. The diterpene esters of Euphorbiaceae latex are lipophilic and non-volatile and would be expected to persist on textile fibers rather than evaporate [12]. A comparable situation is well described for urushiol, the oleoresin of *Toxicodendron* species, which remains active on clothing, tools and equipment long after initial contact and causes recurrent dermatitis in the absence of any further exposure to the plant. Hand-mediated transfer to more susceptible epithelium is also described with urushiol, where lesions may appear at the transferred site while the hands themselves remain unaffected [24]. The mechanism of tissue injury differs since Toxicodendron dermatitis is a type IV hypersensitivity reaction, whereas manchineel produces direct irritation, but the relevant parallel is the physical persistence of a lipophilic plant oleoresin on fabric. The patient developed no dermatitis at any point in her illness, which argues against percutaneous absorption as the route of any re-exposure. Her recurrent symptoms were instead oral and mucosal, and transfer of Euphorbiaceae latex from contaminated hands to a mucosal surface has been described. A woman who pulled a caper spurge (*Euphorbia lathyris*) from her garden bare-handed and subsequently rubbed her eyes developed bilateral conjunctival injection and punctate corneal erosions, without any direct ocular contact with the plant [25]. Such a route would accord with the immediate effect of hydrolysable tannins on oral mucosal protein described above [11].

A clear limitation here is that such an explanation remains an unverifiable hypothesis. The clothing and towel were discarded and so were never available for testing, and no previously published study has measured manchineel toxins on fabric. The stability of these compounds is inferred from work on related Euphorbiaceae species rather than from *Hippomane mancinella* itself. The association reported here is temporal, uncontrolled, and drawn from a single patient. We cannot exclude gradual resolution of a protracted mucosal injury, an attribution effect, or the influence of other avoidance behaviours the patient adopted at the time. Testing the hypothesis requires solvent extraction of the implicated fabric with liquid chromatography–tandem mass spectrometry for hippomanin A and the diterpene esters. Retaining such items for analysis would be worthwhile in future cases. Limitations of our report include: that our patient’s exposure was not toxicologically confirmed, persistence of toxins on fabrics was not analytically demonstrated, symptoms followed a fluctuating course, and cetirizine use could complicate causal interpretation.

Manchineel poisoning is a clinical diagnosis mainly based on exposure history, characteristic symptoms, and exclusion of competing etiologies on the differential diagnosis. There is no confirmatory test available; however, specific tests and characteristics can be used to rule out key mimics such as IgE-mediated anaphylaxis, angioedema, scombroid, and toxicodendron and other phytodermatitis. In contrast to IgE-mediated anaphylaxis, manchineel poisoning is characterized by fruit/tree contact in a tropical setting as opposed to known allergen exposure. While both IgE-mediated anaphylaxis and manchineel poisoning have a rapid onset, manchineel poisoning does not present with urticaria or wheezing. Serum tryptase, which is elevated in anaphylaxis, should be normal or only mildly elevated in manchineel exposure. Manchineel poisoning can be discriminated from angioedema as manchineel poisoning would present with normal trypase, normal C4 and C1-INH levels, lack of ACE inhibitor use, and lack of non-pitting deep dermal/submucosal swelling [2,3,4]. Key characteristics discriminating manchineel poisoning from scombroid include a negative fish ingestion history and lack of a diffuse flushing pattern [26,27]. Manchineel poisoning can be discriminated from toxicodendron and phytodermatitis based on geographical location of exposure and onset. Manchineel is specifically found in tropical coastal areas in the Caribbean, Central and South America, and Southern Florida. Furthermore, contrasting with the slower onset of symptoms in toxicodendron and phytodermatitis, symptoms of manchineel poisoning present within minutes [2,3,4].

The comparators below were selected as foodborne intoxications that a traveller to the same tropical regions might plausibly acquire from an apparently edible food, and are grouped by origin: plant (Table 1) and marine (Table 2). Such a resource is intended to assist front-line clinicians who may encounter ill returned travelers with toxidrome-like symptoms.

**Table 1:** Comparison of select tropical foodborne intoxications of plant origin.

Syndrome	Epidemiology	Etiology	Diagnosis	Treatment	Prevention
**Manchineel toxicity (*Hippomane mancinella*)**	Caribbean, the island of Tobago, Central America, Florida, US Virgin Islands, and the West African coast. Predominantly affects tourists [2,7].	Multiple toxins in sap and fruit, including hippomanin A and phorbol-type diterpene esters, cause irritant dermatitis and mucosal injury [2,7,14].	Clinical diagnosis based on exposure history (sap contact, sheltering under the tree in rain, fruit ingestion): contact dermatitis with blistering, keratoconjunctivitis, or oropharyngeal burning with GI distress after ingestion [1,2,3,4].	Supportive care only. No antidote available. Cutaneous: irrigation with clean water; topical/systemic corticosteroids if severe. Ocular: irrigation with clean water, prompt ophthalmological exam, cycloplegics, topical antibiotics. Ingestion: IV fluids, antihistamines, PPI; monitor for hemodynamic compromise and bradyarrhythmia [2,3,4].	Traveler education: (i.e., avoid trees marked with ‘X’ and do not shelter beneath them in rain; do not eat unfamiliar fruit on beaches). Disposal of clothing and towels potentially exposed to tree sap/fruits may be considered as precautionary measure, if recurrent symptoms are observed [2,3,4].
**Ackee fruit poisoning/Jamaican vomiting sickness (*Blighia sapida*)**	West Africa (origin), the Caribbean (especially Jamaica), Central/South America, and southern Florida. Predominantly affects children and the malnourished; outbreaks documented in Haiti and Burkina Faso [28,29,30].	Hypoglycin A (HGA) in the unripe aril; metabolized to methylenecyclopropylacetic acid, which inhibits fatty-acid β-oxidation, causing hypoketotic hypoglycemia [28,29].	Profuse vomiting altered mental status, and profound hypoglycemia after ingestion of unripe ackee. Supporting labs: hypoglycemia, anion-gap metabolic acidosis, elevated AST; urinary dicarboxylic acids and acylcarnitine profile can confirm [28,29].	Supportive care: IV dextrose, fluid/electrolyte resuscitation, ICU admission for seizures or coma. Riboflavin, glycine, and methylene blue have been proposed but remain unproven [28,31].	Public education to consume only fully ripe ackee; avoid water in which unripe fruit has been boiled; regulatory limits on hypoglycin A in exported canned ackee [28,31].
**Papain-related syndromes (*Carica papaya* enzyme)**	Worldwide; occupational exposure in pharmaceutical, food-processing, textile, and cosmetic industries; non-occupational from meat tenderizers, contact lens cleaners, and topical wound debriders [32,33,34].	Papain, a cysteine protease from papaya latex, is a potent IgE-mediated allergen. Cross-reacts with latex and other fruit cysteine proteases. Spectrum of manifestations: occupational asthma, contact dermatitis, anaphylaxis after ingestion, and anaphylactoid reactions to topical/parenteral exposure [32,33,34].	Clinical diagnosis based on the temporal relationship between exposure and reaction. Specific IgE to papain and skin-prick testing confirm sensitization; serum tryptase may be elevated in anaphylaxis [33,34].	Standard anaphylaxis management: IM epinephrine, H1/H2 antihistamines, corticosteroids, IV fluids, airway support. Occupational asthma: bronchodilators and removal from exposure [33,34].	Avoid papain-containing products in sensitized individuals; read labels on meat tenderizers, supplements, and cosmetics. Occupational engineering controls and respiratory protection. Caution in patients with latex or tropical fruit allergy [32,33].
**Lychee toxicity/lychee-associated acute hypoglycemic encephalopathy (*Litchi chinensis*)**	Lychee-growing regions, notably Muzaffarpur (Bihar, India), Bangladesh, and northern Vietnam. Seasonal outbreaks during harvest (May–June), almost exclusively in undernourished children consuming large amounts on an empty stomach [35,36].	Methylenecyclopropylglycine (MCPG) and hypoglycin A in the aril and seed; homologous to ackee HGA. Disrupt fatty-acid oxidation, producing hypoketotic hypoglycemia, amplified by depleted hepatic glycogen from fasting/malnutrition [35,36].	Sudden-onset early-morning seizures and encephalopathy in a child during harvest. Profound hypoglycemia (often <70 mg/dL), abnormal plasma acylcarnitine profile, and urinary metabolites of HGA and MCPG [35,36].	Immediate IV 10% dextrose; correction of electrolyte derangement; supportive ICU care for seizures and coma. Early glucose correction is lifesaving [35,36].	Limit lychee consumption in young children; ensure an evening meal during harvest season to prevent overnight fasting; community surveillance with rapid dextrose protocols at primary health centers [35,36].
**Cassava poisoning/konzo (*Manihot esculenta*)**	Sub-Saharan Africa (DRC, Mozambique, Tanzania, CAR, Angola, Cameroon, Zambia). Affects hunger-stricken rural populations dependent on bitter cassava; women and children are disproportionately affected. Acute outbreaks have also reported (e.g., Uganda 2017) [37,38,39].	Cyanogenic glycosides (predominantly linamarin) in insufficiently processed cassava; hydrolyzed to release cyanide, which inhibits cytochrome c oxidase. Combined with low sulphur amino acid intake, produces acute cyanide toxicity and chronic neurological disease (konzo, tropical ataxic neuropathy) [37,38].	Acute: cyanide toxidrome (headache, vomiting, tachypnea, lactic acidosis, altered consciousness). Konzo (WHO criteria): symmetric spastic gait, onset within one week in a previously healthy person, exaggerated knee/ankle jerks without spinal disease. Urinary/plasma thiocyanate supports cyanogenic exposure [37,38].	Acute cyanide poisoning: hydroxocobalamin (preferred), sodium thiosulfate, sodium nitrite; oxygen and supportive care. Konzo: no specific treatment; physiotherapy and mobility aids; nutritional support with sulfur amino acid supplementation [37,39].	Adequate cassava processing (wetting method: soaking, fermentation, sun drying, scraping) to reduce cyanogen content. Dietary diversification (protein sources rich in methionine/cysteine). Substitution of sweet (low-cyanogen) cultivars [37,40].

Abbreviations: GI, gastrointestinal; HGA, hypoglycin A; MCPG, methylenecyclopropylglycine; IV, intravenous; PPI, proton pump inhibitor; ICU, intensive care unit; AST, aspartate aminotransferase; WHO, World Health Organization.

**Table 2:** Comparison of select tropical foodborne intoxications of marine origin.

Syndrome	Epidemiology	Etiology	Diagnosis	Treatment	Prevention
**Ciguatera fish poisoning (CFP)**	Most prevalent seafood poisoning worldwide; endemic in tropical/subtropical reef regions (Caribbean, Pacific, Indian Ocean). Increasingly reported in temperate areas with climate change and the global fish trade [41,42].	Ciguatoxins (CTXs) produced by benthic dinoflagellates (*Gambierdiscus* spp.); heat-stable and odorless. Bioaccumulate up the reef food chain, concentrated in large predatory reef fish (barracuda, grouper, snapper, moray eel, amberjack). Toxic effects mediated by activation of voltage-gated sodium channels [41,42,43].	Clinical diagnosis based on reef-fish ingestion: early GI symptoms followed by neurological symptoms of paresthesia, cold allodynia (hot–cold reversal), pruritus, myalgia, arthralgia. Cardiovascular: bradycardia, hypotension. No validated human biomarker [41,42,43].	Supportive care: IV fluids, antiemetics, atropine for bradycardia, vasopressors for hypotension. IV mannitol (1 g/kg) is historically used acutely, though efficacy is debated. Avoid alcohol, nuts, fish, and seafood during convalescence to prevent symptom recurrence. [41,42].	Avoid large predatory reef fish, especially viscera (liver, gonads, roe). Adhere to local advisories; monitor fish supply for regulatory compliance. No cooking method destroys CTX [41,43].
**Scombroid (histamine) fish poisoning**	Worldwide; one of the most common forms of seafood poisoning. Typically sporadic or small clusters linked to a shared meal of improperly refrigerated fish [26,44].	Histamine and other biogenic amines formed by bacterial decarboxylation of histidine in dark flesh of scombroid fish (tuna, mackerel, bonito, skipjack) and non-scombroid species (mahi-mahi, sardine, marlin, anchovy, escolar) stored above 4 °C. Toxin is heat-stable [26,44].	Clinical diagnosis: onset minutes to one hour after ingestion with flushing, urticarial rash, headache, palpitations, diarrhea, oral ‘peppery’ burning; resembles IgE-mediated allergy. Normal serum tryptase distinguishes poisoning from true anaphylaxis. Confirmed by elevated histamine in uneaten fish (>50 mg/100 g) [26,44].	H1 and H2 antihistamines are the mainstay; most cases resolve within 12–48 h. IV fluids; epinephrine and corticosteroids are reserved for severe reactions. Patients on isoniazid or MAOIs may have more severe or prolonged symptoms [26,44].	Refrigerate fish promptly at <4 °C from catch to consumption; regulatory inspection of the cold chain. Counsel patients on isoniazid or MAOIs about higher risk [26,44].
**Neurotoxic shellfish poisoning (NSP)**	Gulf of Mexico (Florida red tide), Caribbean, and New Zealand, associated with *Karenia brevis* blooms. Few annual cases due to monitoring; outbreaks linked to recreationally harvested shellfish. Aerosolized toxins from surf zones cause respiratory illness in beachgoers [27].	Heat-stable Brevetoxins produced by the dinoflagellate *Karenia brevis*, accumulated by filter-feeding bivalves and some gastropods. Toxicity mediated by activation of voltage-gated sodium channels, causing depolarization [27].	Clinical diagnosis based on history of shellfish ingestion within hours of onset: GI symptoms, neurological symptoms (perioral and distal paresthesia, ataxia, slurred speech, hot–cold reversal), and occasionally bronchospasm. No fatalities reported. Brevetoxin metabolite (BTX-3) detectable in urine by LC-MS/MS in research settings [27,45].	Supportive care: IV fluids, antiemetics, monitoring of respiratory and neurological status. Bronchodilators for aerosol-induced bronchospasm. Symptoms typically resolve in 1–72 h [27,46].	Regulatory monitoring of shellfish beds and harvest closures during *K. brevis* blooms; public alerts. Avoid recreational shellfish harvesting in red-tide-affected waters. Susceptible individuals (asthma) should avoid surf zones during blooms [27].
**Diarrhetic shellfish poisoning (DSP)**	Widespread—Europe (leading cause of aquaculture closures), Japan, Chile, South Africa, Australia, and increasingly North America. Outbreaks tied to harmful algal blooms [47,48].	Okadaic acid and dinophysistoxins produced by dinoflagellates (*Dinophysis*, *Prorocentrum*); concentrated by filter-feeding bivalves (mussels, scallops, clams, oysters). Potent inhibitors of protein phosphatases 1 and 2A. Heat- and freeze-stable [47,48].	Clinical diagnosis based of history of bivalve ingestion: nausea, vomiting, severe diarrhea, abdominal cramps, occasionally chills/fever; onset 30 min to a few hours. Confirmation by LC-MS/MS detection of okadaic acid in implicated shellfish [47,48].	Supportive care only: oral or IV rehydration, antiemetics, electrolyte correction. Self-limited, resolving within 72 h; no fatalities reported [47,48].	Routine regulatory shellfish testing with an action limit of 160 µg okadaic acid equivalents per kg tissue; harvest area closures during blooms; consumer adherence to advisories [47,48].
**Tetrodotoxin (TTX) poisoning**	Most cases in Japan, China, Korea, Taiwan, Bangladesh, and Southeast Asia, where pufferfish (fugu) is consumed. Sporadic cases in the Mediterranean (invasive *Lagocephalus sceleratus*), Caribbean, and the US. Mortality up to 10–15% in some series [49,50].	Heat-stable tetrodotoxin is produced by symbiotic bacteria; concentrated in the viscera (liver, gonads, skin) of pufferfish, porcupine fish, ocean sunfish, blue-ringed octopus, certain newts, and some marine gastropods. Potent voltage-gated sodium-channel blocker [49,50].	Clinical diagnosis based on ingestion history and progressive pattern of toxicity: onset within minutes to hours with perioral paresthesia ascending to facial and limb numbness, dysarthria, weakness, ascending paralysis, hypotension, bradycardia, and respiratory failure; consciousness preserved in mild cases. TTX detectable in serum/urine by LC-MS/MS where available [49,50].	No specific antidote. Aggressive supportive care: airway protection and mechanical ventilation for respiratory failure, IV fluids and vasopressors for hypotension, atropine for bradycardia. Activated charcoal/gastric lavage if early presentation [49,50].	Strict regulation of fugu preparation (licensed chefs only in Japan); public education to avoid pufferfish from unregulated sources; awareness of invasive *Lagocephalus sceleratus* in the Mediterranean. No safe culinary method removes TTX from viscera [49,50,51].

Abbreviations: CFP, ciguatera fish poisoning; NSP, neurotoxic shellfish poisoning; DSP, diarrhetic shellfish poisoning; TTX, tetrodotoxin; CTX, ciguatoxin; GI, gastrointestinal; IV, intravenous; LC-MS/MS, liquid chromatography–tandem mass spectrometry; MAOI, monoamine oxidase inhibitor; H1/H2, histamine receptor types 1 and 2.

### Comparison of Common Tropical Foodborne Intoxication Syndromes with Manchineel Toxicity

Manchineel toxicity is mediated by the inflammatory and irritant effects of phorbol-ester-rich sap and fruit on the skin, mucous membranes, and gut. Manchineel toxicity is predominantly a local irritant phenomenon, and the rare hemodynamic effects reported after ingestion remain mechanistically unexplained, while other common plant-food intoxications are caused by ingestion of plant tissues that harbour metabolic, cyanogenic, allergenic, or neurotoxic principles [2,14]. Ackee (*Blighia sapida*) and lychee (*Litchi chinensis*) both contain hypoglycin A (lychee also contains methylenecyclopropylglycine), which inhibits fatty-acid β-oxidation and produces a Reye-like syndrome of vomiting, hypoketotic hypoglycemia, and encephalopathy in undernourished children. This is an ingestion-dependent metabolic emergency in which intravenous dextrose is life saving, whereas manchineel toxicity is only treated with supportive care [28,29,35,36]. Cassava and konzo (*Manihot esculenta*) release cyanide from linamarin, which inhibits cytochrome c oxidase and leads to an acute cyanide toxidrome or chronic upper-motor-neuron disease in sub-Saharan subsistence populations [37,38,39]. Papain (*Carica papaya*) is an IgE-mediated cysteine-protease allergen causing occupational asthma, contact dermatitis, and anaphylaxis. Papain has significant latex cross-reactivity. Papain-related syndromes are managed by allergen avoidance and standard anaphylaxis protocols [32,33,34].

Across these comparators, the key distinctions from manchineel are the route of injury (ingestion rather than direct tree contact), the populations affected (most often children or the malnourished rather than tourists), and the availability of specific dietary, regulatory, or processing-based prevention strategies.

## 4. Conclusions

The manchineel tree is a highly toxic tree, which causes severe contact dermatitis upon cutaneous exposures to the sap, tree components, or contaminated rainwater, as well as oral and gastric manifestations from ingestion of the fruit. Travellers to regions in the Caribbean and Central America are at particular risk if unfamiliar with the associated dangers. They should be counselled to avoid the tree and unfamiliar fruit found on the ground. Similarly, travellers should be cautioned to avoid sheltering from rain underneath trees resembling the manchineel tree, or in areas of beaches clearly marked with an ‘X’. When exposures occur, supportive care may help with symptomatic relief, and it may be reasonable to dispose of potentially contaminated clothing, particularly if recurrent symptoms are observed, as in our patient.

## Data Availability

All available data are presented in the manuscript.
